# Management of Stable Angina with Ivabradine as Safe Alternative to Patients with Myasthenia Gravis

**DOI:** 10.1155/2016/3589204

**Published:** 2016-10-16

**Authors:** Giuliano Ohde Dalledone, Gustavo Lenci Marques, Renata Dal-Prá Ducci, Arnaldo Laffitte Stier Junior, Cláudia Suemi Kamoi Kay, Lineu Cesar Werneck, Paulo José Lorenzoni, Rosana Herminia Scola

**Affiliations:** ^1^Service of Cardiology, Department of Internal Medicine, Hospital de Clínicas, Universidade Federal do Paraná (UFPR), Curitiba, PR, Brazil; ^2^Service of Neuromuscular Disorders, Division of Neurology, Department of Internal Medicine, Hospital de Clínicas, Universidade Federal do Paraná (UFPR), Curitiba, PR, Brazil

## Abstract

Management of cardiac symptoms in myasthenia gravis (MG) patients can be challenging. The aim of this report is to describe the safe use of ivabradine for stable angina in MG patients. A 48 y.o. woman, with MG diagnosis, presented stable angina. Therapies choices were reduced considering concomitant disease as well as previous and unsuccessful cardiologic managements. Ivabradine showed unexpected results. The patient presented an improvement of neurological and cardiac symptoms, bringing ivabradine as one more therapeutic option to similar patients. In this report we recommend ivabradine as an effective and safe drug for treatment of stable angina in MG patients.

## 1. Introduction

Several cardiac disorders could develop in patients with myasthenia gravis (MG) [1]. Similar to that recommended to the normal population, therapy for cardiac conditions must be provided to decrease the risk of death by cardiovascular event in these patients.

In this situation, cardiac management with conventional drugs can be difficult because some of them can induce a deterioration of MG or even a myasthenic crisis. Hence, plenty of cardiologic drugs, as beta-blockers, are contraindicated for MG patients which makes cardiologic management much more complicated [2]. In addition, there are many studies and reports that deal only with the effects of first-generation cardiologic drugs on the neuromuscular junction and the risk of these drugs for MG patients. Therefore, it makes sense to report on the safety of the new cardiologic therapies for MG patients.

The aim of this study is to describe the safety and beneficial use of ivabradine on remission of stable angina in an MG patient.

## 2. Case Report

A 48-year-old woman, diagnosed with myasthenia gravis, with positive serum anti-acetylcholine receptor antibody and decremental response after repetitive nerve stimulation, which was partially controlled with sporadic use of pyridostigmine and prior thymectomy, had an acute myocardial infarction (AMI). She also was previously reported due to coexistence of other immune-mediated disorders [3], and, in the recent years, she also developed Sjögren's syndrome and neuromyelitis optica spectrum disorder. At the moment, she is independent for daily activities and stable for all concomitant disorders.

**Figure 1:**
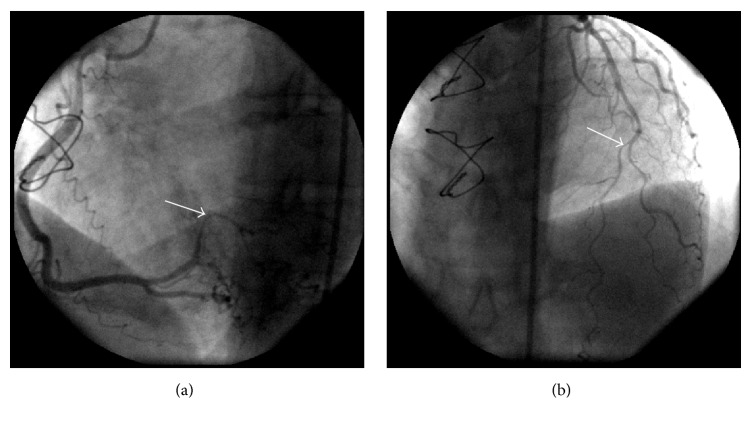
Coronary angiography showed posterior branch of the right coronary artery with a severe stenosis (90%) (a) and medium segment of the anterior descendent artery suboccluded (70%) (b).

She suffered an episode of typical angina and electrocardiography (ECG) showed no ST-segment elevation, but T waves were inverted in DII, DIII, aVF, V4, V5, and V6 derivations and serum troponin and CK-MB dosages were elevated. Initial AMI standard therapy was started and she also underwent percutaneous coronary angiography, which revealed severe stenosis of the posterior branch (90%) of the right coronary artery and medium segment of the anterior descendent artery (70%) (Figure 1). Moreover, ventriculography exam showed low contractibility on inferoapical wall of her left ventricle. Stenting and surgery were not possible due to the coronary anatomy being unsuitable. Her adjuvant therapy was enalapril, simvastatin, aspirin, and clopidogrel. After the acute phase, clopidogrel was suspended. She had no history of hypertension, hypercholesterolemia, diabetes mellitus, smoking, or drinking, and there was no history of ischemic heart disease in her family.

Few years after the AMI, she began to have frequent episodes of angina pectoris after strenuous exercise as well as palpitations, and then isosorbide mononitrate (20 mg t.i.d.) was administered. At this point, 12-lead ECG records showed 68 beats/min, normal sinus rhythm, and inverted T waves in DII, DIII, aVF, V4, V5, and V6 derivations; and an echocardiogram showed 69% ejection fraction and normal left ventricular systolic function without wall motion abnormality or significant valvular abnormality. However, no cardiologic beneficial effect was observed after 3 months and she continued to experience a worsening frequency and intensity of symptoms (angina pectoris at rest). After an appointment with another service, isosorbide mononitrate was withdrawn and metoprolol was prescribed, which improved her angina but caused weakness and dyspnea due to the exacerbation of MG symptoms. After metoprolol was stopped, her MG symptoms improved, but her cardiologic symptoms returned and progressively worsened (Canadian Cardiovascular Society grading of angina pectoris score; CCS score: class 2).

**Figure 2:**
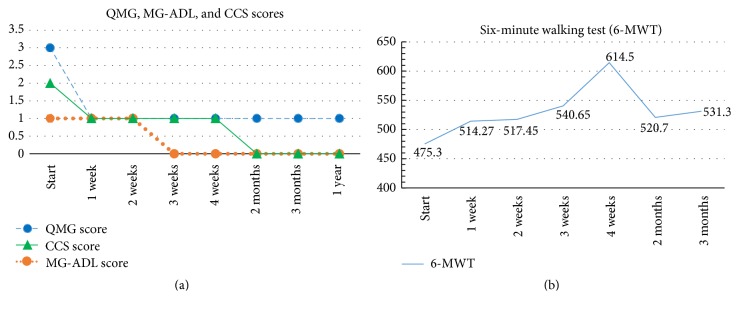
(a) Graphic shows QMG, MG-ADL, and CCS scores before and during the initial 12 months of therapy. (b) Graphic shows 6-MWT scores before and during the initial 4 weeks of therapy. (QMG: Quantitative Myasthenia Gravis test; MG-ADL: Myasthenia Gravis-specific Activities of Daily Living scale; CCS: Canadian Cardiovascular Society grading of angina pectoris; 6-MWT: 6-minute walking test.)

Therewith, we opted to use ivabradine (5 mg b.i.d.) to treat her stable angina. We checked her Quantitative Myasthenia Gravis test (QMG), Myasthenia Gravis-specific Activities of Daily Living scale (MG-ADL), and the CCS score before therapy and at regular intervals over 1 year and the 6-minute walking test (6-MWT) was also performed once a week for 4 weeks (Figure 2). Significant improvement of the cardiac symptoms was observed, with angina persisting only after strenuous exercises (CCS class 1). Ivabradine dosage was increased (7.5 mg b.i.d.) which resulted in the patient being asymptomatic after one month. The MG symptoms were not exacerbated during this new treatment.

All studies were performed after the consent of the patient.

## 3. Discussion

Stable angina management focuses on symptom extinction [4]. Drug therapy for stable angina includes antiplatelet agents, lipid-lowering drugs, beta-blockers, and angiotensin I-converting enzyme (ACE) inhibitors, which help decrease the incidence of myocardium ischemia and increase patient survival [4]. In addition, nitrates, calcium-channel blockers, trimetazidine, and ivabradine decrease the symptoms and episodes of myocardial ischemia, improving the patient's quality of life [4]. According to cardiologic guidelines, stable angina therapy should be initiated with these medications, which can decrease morbidity and mortality and, when necessary, combine them with medications to control angina and decrease myocardial ischemia [4]. However, many of these drugs have the potential to exacerbate MG symptoms and are contraindicated or should be used with caution by MG patients. The literature is limited to report the use of cardiologic medications in MG patients.

Nitrates are safe for use by MG patients, since there are no reports contraindicating the use of nitrates (fast-acting or long-acting) in MG patients, but unfortunately this drug had no beneficial response in our patient. Metoprolol provided an improvement in her angina symptoms, but this therapy was not tolerated because it exacerbated the patient's MG symptoms. Indeed, beta-blockers and calcium-channel blockers have been described as causing a worsening of MG symptoms. Trimetazidine was also reported to cause a neuromuscular junction transmission defect in experimental animal models [5]. Therefore, it makes sense to avoid this drug in treating these patients.

Ivabradine is a new drug which acts by inhibiting the sinoatrial node funny (I_f_) current by selectively blocking HCN (hyperpolarization-activated, cyclonucleotide-gated) ion channels [6]. These channels are responsible for the I_f_ current and have four distinct isoforms, which are expressed in the heart, retina, and brain [6]. Therefore, because HCN channels are not expressed in other systems, it is probable that their inhibition does not interfere in muscle contraction or neuromuscular transmission. Indeed, the blockage of I_f_ channels causes a reduction in myocardial oxygen demand without changing cardiac inotropism or arterial pressure [6]. So, ivabradine has been indicated for use in the treatment of stable angina or when there are contraindications to using beta-blockers, such as in MG patients. Although these findings should be confirmation in larger series, we believe that our report contributes to affirming that ivabradine can be considered an effective and safe drug option for the treatment of stable angina in MG patients.
